# Supplementary material for: A Likelihood Approach for Real-Time Calibration of Stochastic Compartmental Epidemic Models
Source: PLoS Comput Biol. 2017 Jan 17;13(1):e1005257. doi: 10.1371/journal.pcbi.1005257 (PMC5240920; doi:10.1371/journal.pcbi.1005257)
Supplement: S1 File — (TAR.GZ) [file pcbi.1005257.s014.tar.gz › HSPH_Online-SI-Revision/Additions/IPoi10/n100-extreme-SEITR/n100-extreme-SEITR_BENCH2_table0.pdf]

1	newly 3 weeks spec	6372.	6000.66	{3594., 9162.}	28.7374	0.135546	newly 3 weeks cumul	27578.	21395.7	{13343., 30167.}	26.0834	0.143071	newly 5 weeks spec	2747.	3875.92	{2039., 6353.}	54.7947	0.17566	newly 5 weeks cumul	34544.	30144.8	{18797., 44021.}	26.1724	0.13229	newly 51 weeks cumul	38811.	42765.5	{24855., 65428.}	33.1135	0.133326
2	newly 3 weeks spec	7321.	9716.48	{5632., 15886.}	43.3154	0.149069	newly 3 weeks cumul	36564.	36774.	{26470., 45191.}	16.5383	0.0744125	newly 5 weeks spec	2664.	4612.79	{2305., 8775.}	78.7846	0.216853	newly 5 weeks cumul	43633.	48258.4	{32475., 66017.}	24.0802	0.0980916	newly 58 weeks cumul	47260.	56326.	{36300., 82110.}	30.6025	0.115711
3	newly 3 weeks spec	8747.	4265.16	{2474., 6263.}	53.1757	0.354457	newly 3 weeks cumul	40258.	23258.3	{14251., 33412.}	44.1307	0.270815	newly 5 weeks spec	3413.	1359.09	{723., 2006.}	60.8685	0.441682	newly 5 weeks cumul	49075.	27043.9	{16282., 38945.}	46.6176	0.292277	newly 60 weeks cumul	54208.	28728.1	{17174., 41366.}	48.411	0.308753
4	newly 3 weeks spec	8496.	5114.71	{2876., 8195.}	43.0995	0.269336	newly 3 weeks cumul	36998.	23778.1	{14424., 34933.}	37.5273	0.226301	newly 5 weeks spec	3304.	2244.44	{1082., 3278.}	51.4547	0.293484	newly 5 weeks cumul	45863.	29386.9	{17723., 43709.}	38.1578	0.230267	newly 58 weeks cumul	50745.	33226.6	{19309., 50848.}	39.7604	0.23737
5	newly 3 weeks spec	5120.	7853.99	{4924., 12888.}	57.4722	0.17223	newly 3 weeks cumul	31803.	37185.7	{26843., 52852.}	26.2311	0.0993829	newly 5 weeks spec	1318.	2649.45	{1544., 4486.}	102.042	0.261707	newly 5 weeks cumul	35687.	44486.4	{31158., 65283.}	32.2253	0.115012	newly 60 weeks cumul	37045.	47807.6	{33115., 71068.}	35.5492	0.123178
6	newly 3 weeks spec	4778.	7903.33	{4043., 14584.}	72.0538	0.203376	newly 3 weeks cumul	31868.	34134.9	{21511., 51272.}	27.5395	0.114733	newly 5 weeks spec	1057.	3178.63	{1350., 6147.}	201.168	0.391253	newly 5 weeks cumul	35197.	42455.2	{25327., 67069.}	36.2306	0.134522	newly 59 weeks cumul	35989.	47023.8	{27190., 76603.}	43.3627	0.148928
7	newly 3 weeks spec	7357.	5595.71	{3413., 8237.}	29.8406	0.163147	newly 3 weeks cumul	28195.	20494.6	{13601., 27874.}	27.926	0.155014	newly 5 weeks spec	3977.	3491.41	{1977., 5186.}	30.9088	0.151699	newly 5 weeks cumul	37670.	28445.2	{18223., 39971.}	27.9753	0.153414	newly 51 weeks cumul	47209.	39426.7	{24248., 56175.}	27.44	0.141045
8	newly 3 weeks spec	7216.	7263.83	{4025., 12429.}	37.8138	0.165601	newly 3 weeks cumul	32722.	31724.	{20753., 46923.}	26.7488	0.122294	newly 5 weeks spec	3068.	2885.27	{1519., 5402.}	41.968	0.196128	newly 5 weeks cumul	40537.	39260.5	{24830., 60627.}	29.4407	0.134298	newly 58 weeks cumul	46063.	43675.7	{26967., 68800.}	30.7493	0.142925
9	newly 3 weeks spec	5164.	8786.07	{4616., 15490.}	75.0568	0.208649	newly 3 weeks cumul	32212.	39031.8	{25844., 55269.}	31.6054	0.116715	newly 5 weeks spec	1330.	3128.59	{1455., 5888.}	135.871	0.30546	newly 5 weeks cumul	36196.	47531.9	{30046., 71169.}	40.0828	0.137902	newly 60 weeks cumul	37584.	51518.5	{31914., 78910.}	44.8488	0.148723
10	newly 3 weeks spec	8694.	5807.67	{2853., 11333.}	43.2579	0.262043	newly 3 weeks cumul	33638.	22996.5	{13099., 38199.}	35.312	0.209024	newly 5 weeks spec	4707.	2989.19	{1299., 6155.}	50.4504	0.314336	newly 5 weeks cumul	44803.	30219.3	{16335., 53231.}	38.3353	0.229505	newly 56 weeks cumul	56292.	36595.9	{19008., 66774.}	41.8479	0.255255
11	newly 3 weeks spec	8565.	6953.3	{3988., 10907.}	32.5277	0.174555	newly 3 weeks cumul	34772.	25869.8	{17166., 35160.}	26.7327	0.150427	newly 5 weeks spec	4255.	3982.47	{2030., 6283.}	41.763	0.19451	newly 5 weeks cumul	45069.	35209.3	{22049., 50263.}	28.2058	0.155548	newly 55 weeks cumul	54734.	45017.9	{26717., 67873.}	31.2613	0.16511
12	newly 3 weeks spec	7136.	7201.39	{4306., 10479.}	27.8791	0.122787	newly 3 weeks cumul	31568.	28392.1	{19442., 37322.}	19.3115	0.0968309	newly 5 weeks spec	3139.	3711.33	{1924., 5297.}	41.2059	0.145716	newly 5 weeks cumul	39318.	37368.5	{24242., 51932.}	22.2816	0.10569	newly 56 weeks cumul	44760.	45063.3	{28061., 62871.}	25.6033	0.112822
13	newly 3 weeks spec	7637.	6041.97	{3486., 10053.}	37.3939	0.197634	newly 3 weeks cumul	33066.	25471.2	{16570., 38148.}	30.1993	0.163325	newly 5 weeks spec	3594.	2809.61	{1446., 4698.}	44.8339	0.235304	newly 5 weeks cumul	41882.	32474.9	{20311., 51218.}	32.6783	0.175861	newly 57 weeks cumul	49021.	37740.2	{23028., 60028.}	34.9966	0.188283
14	newly 3 weeks spec	8364.	5374.55	{2752., 9463.}	40.0009	0.250132	newly 3 weeks cumul	30226.	18555.	{10914., 27782.}	38.6125	0.237224	newly 5 weeks spec	4850.	3704.05	{1680., 7226.}	46.573	0.262396	newly 5 weeks cumul	41750.	26769.4	{14743., 44163.}	37.6621	0.233408	newly 50 weeks cumul	54343.	40026.1	{19927., 71513.}	40.4775	0.233134
15	newly 3 weeks spec	7352.	5862.32	{3816., 8143.}	27.7292	0.145602	newly 3 weeks cumul	31019.	21255.6	{13692., 28270.}	31.4757	0.176841	newly 5 weeks spec	3380.	3744.2	{2215., 5570.}	31.0168	0.119322	newly 5 weeks cumul	39368.	29706.3	{19866., 40436.}	27.5155	0.149016	newly 51 weeks cumul	45493.	41875.9	{26473., 59987.}	25.8982	0.121299
16	newly 3 weeks spec	8547.	5232.61	{2671., 10583.}	47.6677	0.297225	newly 3 weeks cumul	32656.	19999.	{11876., 34466.}	40.6719	0.250697	newly 5 weeks spec	4015.	2948.51	{1408., 6237.}	51.0073	0.283658	newly 5 weeks cumul	42788.	26914.3	{15217., 50065.}	42.9246	0.262125	newly 54 weeks cumul	49793.	34225.8	{18606., 66283.}	43.8557	0.254495
17	newly 3 weeks spec	6343.	8339.44	{4745., 12953.}	45.4696	0.153148	newly 3 weeks cumul	39322.	38718.4	{26730., 53086.}	22.7284	0.102671	newly 5 weeks spec	1376.	2845.26	{1556., 4695.}	108.303	0.274882	newly 5 weeks cumul	43673.	46538.6	{31176., 65681.}	26.4073	0.109687	newly 60 weeks cumul	44678.	50134.2	{33163., 71435.}	29.1833	0.114963
18	newly 3 weeks spec	6778.	7311.63	{3954., 12505.}	37.7725	0.149768	newly 3 weeks cumul	35236.	33115.8	{21070., 48371.}	24.0596	0.112675	newly 5 weeks spec	2583.	2751.34	{1342., 4856.}	44.33	0.175044	newly 5 weeks cumul	42014.	40443.3	{24772., 62917.}	27.1644	0.123299	newly 59 weeks cumul	45747.	44257.3	{26680., 69853.}	28.6564	0.12882
19	newly 3 weeks spec	8875.	7434.35	{4640., 10200.}	25.1619	0.130456	newly 3 weeks cumul	35227.	27301.2	{18945., 34707.}	22.6689	0.122395	newly 5 weeks spec	4524.	4347.72	{2442., 6250.}	31.5998	0.142779	newly 5 weeks cumul	46101.	37435.3	{25004., 49465.}	22.5033	0.11878	newly 54 weeks cumul	56361.	48678.4	{30684., 65685.}	24.242	0.122404
20	newly 3 weeks spec	6233.	7089.14	{4114., 12359.}	35.2937	0.127749	newly 3 weeks cumul	33857.	31899.9	{21694., 48072.}	21.9965	0.100656	newly 5 weeks spec	2013.	2865.22	{1448., 4773.}	58.9605	0.154047	newly 5 weeks cumul	39540.	39369.8	{25926., 61340.}	24.8339	0.106806	newly 59 weeks cumul	42220.	43504.8	{27898., 68134.}	27.3828	0.112163
21	newly 3 weeks spec	6457.	8009.73	{4136., 15050.}	44.9618	0.155979	newly 3 weeks cumul	36224.	36879.9	{23197., 55059.}	24.9741	0.108364	newly 5 weeks spec	1848.	2762.97	{1281., 5488.}	63.3318	0.183692	newly 5 weeks cumul	41535.	44435.5	{26781., 69980.}	28.8618	0.118263	newly 60 weeks cumul	43675.	47925.1	{28367., 77148.}	30.7029	0.122221
22	newly 3 weeks spec	7395.	4478.35	{1932., 8114.}	47.4538	0.307609	newly 3 weeks cumul	32127.	19277.8	{9171., 33393.}	43.7776	0.282222	newly 5 weeks spec	2882.	2089.96	{866., 3846.}	46.9685	0.273728	newly 5 weeks cumul	39687.	24459.	{11271., 43061.}	44.1487	0.281965	newly 56 weeks cumul	44378.	28603.5	{12876., 50920.}	43.7565	0.274002
23	newly 3 weeks spec	4881.	8098.19	{4410., 13235.}	69.6713	0.202796	newly 3 weeks cumul	26601.	31627.	{20290., 43223.}	30.4918	0.115633	newly 5 weeks spec	1370.	3968.82	{1865., 6823.}	189.695	0.403055	newly 5 weeks cumul	30613.	41387.7	{24858., 61088.}	42.8152	0.146114	newly 57 weeks cumul	31913.	48998.9	{28305., 74032.}	57.4811	0.178103
24	newly 3 weeks spec	7357.	10921.5	{6445., 15557.}	54.263	0.177229	newly 3 weeks cumul	40276.	40745.6	{30513., 48565.}	15.0715	0.0677937	newly 5 weeks spec	2182.	5024.56	{2507., 7670.}	132.257	0.319807	newly 5 weeks cumul	46540.	53429.	{37491., 65708.}	24.1598	0.0966525	newly 59 weeks cumul	48960.	61384.1	{41189., 79894.}	32.9768	0.122117
25	newly 3 weeks spec	9005.	8769.23	{6003., 12440.}	21.6337	0.0979818	newly 3 weeks cumul	36976.	30742.5	{24002., 37204.}	17.209	0.0898944	newly 5 weeks spec	3489.	5346.26	{3137., 9036.}	58.3253	0.173162	newly 5 weeks cumul	46402.	43061.3	{32417., 55675.}	16.6378	0.0792877	newly 55 weeks cumul	51783.	56806.8	{39768., 80724.}	24.0516	0.0963673
26	newly 3 weeks spec	6466.	8043.34	{5321., 11386.}	32.2337	0.11779	newly 3 weeks cumul	27532.	27643.5	{20796., 33505.}	15.6714	0.0723314	newly 5 weeks spec	2648.	5204.59	{2979., 8331.}	97.3063	0.263236	newly 5 weeks cumul	34415.	39394.	{27617., 51394.}	23.255	0.0914213	newly 53 weeks cumul	38357.	54742.2	{35758., 75893.}	45.7585	0.152083
27	newly 3 weeks spec	7481.	5080.74	{2877., 8256.}	38.974	0.227859	newly 3 weeks cumul	29075.	19469.9	{12000., 29539.}	34.8105	0.204085	newly 5 weeks spec	4125.	2886.82	{1519., 4910.}	42.9554	0.24585	newly 5 weeks cumul	38835.	26222.2	{15480., 41094.}	36.5324	0.213802	newly 53 weeks cumul	49190.	33994.8	{19288., 54453.}	38.1725	0.220322
28	newly 3 weeks spec	5303.	4306.59	{2515., 8571.}	38.9863	0.196899	newly 3 weeks cumul	34205.	23260.6	{15402., 41940.}	39.3833	0.22385	newly 5 weeks spec	965.	1378.35	{808., 2837.}	52.545	0.155188	newly 5 weeks cumul	37460.	27103.9	{17712., 49734.}	38.1318	0.208998	newly 60 weeks cumul	38276.	28816.5	{18826., 53227.}	37.0789	0.19824
29	newly 3 weeks spec	9186.	3705.76	{2023., 6585.}	60.3075	0.442529	newly 3 weeks cumul	36850.	16723.6	{9683., 26261.}	55.0905	0.380157	newly 5 weeks spec	4278.	1797.6	{822., 2948.}	67.1785	0.488882	newly 5 weeks cumul	47405.	21098.8	{11789., 36586.}	56.0446	0.393318	newly 57 weeks cumul	55913.	24643.5	{13265., 41864.}	58.4366	0.413346
30	newly 3 weeks spec	9408.	11130.3	{6641., 18770.}	37.6157	0.141564	newly 3 weeks cumul	43928.	41248.7	{30406., 51191.}	15.8683	0.0806947	newly 5 weeks spec	3519.	5340.96	{2363., 11698.}	73.1264	0.20581	newly 5 weeks cumul	53150.	54599.9	{39831., 71993.}	19.7326	0.0858225	newly 60 weeks cumul	58223.	62863.5	{43246., 89439.}	25.1117	0.103446
31	newly 3 weeks spec	6334.	5550.05	{3658., 8528.}	27.0596	0.133076	newly 3 weeks cumul	24601.	19769.5	{13941., 27282.}	24.2733	0.12735	newly 5 weeks spec	3288.	3690.89	{2173., 5858.}	36.1604	0.135197	newly 5 weeks cumul	32650.	28034.9	{19222., 41226.}	24.6797	0.123638	newly 50 weeks cumul	39652.	40828.2	{26274., 61265.}	28.4074	0.118329
32	newly 3 weeks spec	8242.	6497.94	{3793., 9853.}	31.2524	0.170675	newly 3 weeks cumul	32101.	26078.7	{17021., 36804.}	24.7204	0.132051	newly 5 weeks spec	4308.	3305.57	{1772., 5036.}	37.8758	0.206583	newly 5 weeks cumul	42469.	34084.7	{21400., 49255.}	27.0157	0.145799	newly 56 weeks cumul	52787.	41117.6	{24939., 60108.}	29.9104	0.163676
33	newly 3 weeks spec	5633.	1834.23	{767., 2864.}	70.9482	0.57562	newly 3 weeks cumul	30340.	11106.8	{4943., 17667.}	66.2873	0.50919	newly 5 weeks spec	2012.	529.657	{219., 826.}	75.9841	0.67234	newly 5 weeks cumul	35798.	12623.4	{5522., 20038.}	67.5511	0.527351	newly 60 weeks cumul	38479.	13211.8	{5778., 20935.}	68.2871	0.539003
34	newly 3 weeks spec	6383.	5016.1	{2719., 7748.}	40.7424	0.209833	newly 3 weeks cumul	29792.	20973.9	{12592., 32101.}	36.2125	0.203274	newly 5 weeks spec	2446.	2414.97	{1222., 3594.}	43.5206	0.178479	newly 5 weeks cumul	36225.	26912.1	{15635., 41071.}	37.101	0.200651	newly 56 weeks cumul	39694.	31758.1	{17984., 48138.}	37.0433	0.189254
35	newly 3 weeks spec	5611.	7839.11	{4267., 11969.}	51.6664	0.16681	newly 3 weeks cumul	36155.	37017.3	{23522., 51161.}	25.0199	0.109718	newly 5 weeks spec	1151.	2677.4	{1315., 4214.}	132.778	0.314974	newly 5 weeks cumul	39920.	44387.	{27132., 62671.}	29.9896	0.12071	newly 60 weeks cumul	40865.	47767.1	{28708., 68116.}	33.2568	0.12732
36	newly 3 weeks spec	9648.	9400.12	{5885., 13335.}	24.0929	0.11083	newly 3 weeks cumul	37219.	32630.7	{24715., 39199.}	14.8723	0.0765395	newly 5 weeks spec	5029.	5542.58	{2941., 8936.}	40.0514	0.160381	newly 5 weeks cumul	49093.	45573.1	{32284., 58306.}	17.4697	0.0841576	newly 56 weeks cumul	60543.	58308.	{38241., 79181.}	22.6752	0.104974
37	newly 3 weeks spec	8031.	9167.77	{6094., 13672.}	28.2645	0.10871	newly 3 weeks cumul	39903.	33710.3	{26043., 41647.}	17.2221	0.0881857	newly 5 weeks spec	2840.	4963.62	{2853., 8364.}	76.7099	0.20902	newly 5 weeks cumul	47625.	45584.8	{33290., 59441.}	18.0718	0.0831391	newly 57 weeks cumul	51821.	56177.5	{38732., 79632.}	23.9002	0.0962805
38	newly 3 weeks spec	6443.	7049.35	{3637., 12022.}	35.4233	0.145873	newly 3 weeks cumul	29746.	25355.5	{15708., 34777.}	21.9717	0.115417	newly 5 weeks spec	2643.	4207.08	{1843., 8592.}	72.7117	0.210036	newly 5 weeks cumul	36633.	35123.9	{20149., 53173.}	26.7686	0.12628	newly 54 weeks cumul	40381.	45940.8	{24539., 77162.}	36.0926	0.142125
39	newly 3 weeks spec	8056.	7206.41	{4280., 12281.}	33.8835	0.164956	newly 3 weeks cumul	34505.	27486.2	{18649., 39763.}	26.6383	0.142165	newly 5 weeks spec	3317.	3895.13	{1967., 7324.}	48.066	0.175314	newly 5 weeks cumul	43007.	36764.9	{23789., 56303.}	28.3114	0.143576	newly 56 weeks cumul	48356.	45500.7	{28008., 73968.}	31.7202	0.147145
40	newly 3 weeks spec	7422.	3900.56	{2058., 6224.}	49.8185	0.331282	newly 3 weeks cumul	30770.	17909.2	{9930., 27073.}	43.7902	0.276988	newly 5 weeks spec	3674.	1676.91	{864., 2642.}	56.373	0.397597	newly 5 weeks cumul	39721.	22166.6	{12151., 33763.}	46.184	0.297583	newly 57 weeks cumul	48338.	25227.1	{13641., 38554.}	49.3789	0.326749
41	newly 3 weeks spec	7711.	11530.5	{6192., 19256.}	57.8057	0.182391	newly 3 weeks cumul	45986.	42300.1	{31072., 51384.}	14.3421	0.0715798	newly 5 weeks spec	1635.	5183.11	{2182., 10884.}	217.01	0.424821	newly 5 weeks cumul	51212.	55558.8	{37015., 72164.}	22.0422	0.0916391	newly 60 weeks cumul	52636.	63241.	{39918., 89278.}	31.0142	0.117075
42	newly 3 weeks spec	7808.	5861.1	{3491., 9207.}	32.7558	0.18221	newly 3 weeks cumul	27957.	21080.6	{13859., 29840.}	27.3703	0.151208	newly 5 weeks spec	4650.	3682.35	{1967., 6025.}	36.7824	0.197531	newly 5 weeks cumul	38862.	29468.6	{18533., 43501.}	29.7873	0.164281	newly 51 weeks cumul	51353.	41129.8	{24578., 63987.}	31.5034	0.167767
43	newly 3 weeks spec	8961.	5244.	{2887., 8384.}	43.8272	0.275851	newly 3 weeks cumul	34781.	21754.2	{12742., 32601.}	38.1635	0.230471	newly 5 weeks spec	4598.	2548.96	{1269., 4202.}	47.9259	0.311737	newly 5 weeks cumul	46123.	27995.7	{15946., 42802.}	40.5414	0.248951	newly 56 weeks cumul	56656.	33290.2	{18540., 51858.}	42.7546	0.266916
44	newly 3 weeks spec	7188.	8515.63	{4977., 13718.}	32.9785	0.124613	newly 3 weeks cumul	31836.	31446.8	{21405., 39747.}	16.3054	0.0748169	newly 5 weeks spec	3246.	4612.12	{2346., 8256.}	53.5343	0.166946	newly 5 weeks cumul	39959.	42426.4	{27218., 59457.}	22.2925	0.0944082	newly 56 weeks cumul	46072.	52396.1	{31909., 78364.}	28.24	0.110675
45	newly 3 weeks spec	7528.	7817.91	{3893., 14735.}	36.5485	0.156213	newly 3 weeks cumul	33385.	32729.9	{20135., 47939.}	24.1065	0.110925	newly 5 weeks spec	3187.	3423.75	{1452., 6840.}	49.3451	0.196132	newly 5 weeks cumul	41374.	41440.1	{24207., 66330.}	27.3317	0.122434	newly 58 weeks cumul	46827.	46814.7	{26381., 77631.}	29.792	0.132601
46	newly 3 weeks spec	8312.	2824.12	{1412., 4859.}	67.751	0.543734	newly 3 weeks cumul	39284.	15752.3	{8315., 26899.}	61.1754	0.455281	newly 5 weeks spec	3124.	888.075	{426., 1510.}	73.0182	0.62939	newly 5 weeks cumul	47611.	18236.3	{9470., 31198.}	62.9819	0.477858	newly 60 weeks cumul	52255.	19337.2	{10011., 33020.}	64.1834	0.493651
47	newly 3 weeks spec	8223.	6873.27	{4101., 10024.}	29.4944	0.153687	newly 3 weeks cumul	34349.	26118.5	{16260., 35928.}	25.9732	0.14536	newly 5 weeks spec	3657.	3870.28	{1920., 5843.}	39.7688	0.163897	newly 5 weeks cumul	43642.	35207.	{21793., 49266.}	26.1683	0.140009	newly 55 weeks cumul	49521.	44818.4	{26201., 63786.}	28.5531	0.138989
48	newly 3 weeks spec	8405.	7883.89	{4732., 10906.}	25.9679	0.121904	newly 3 weeks cumul	39417.	31542.4	{21772., 40478.}	21.7214	0.115251	newly 5 weeks spec	2763.	3900.31	{1993., 5441.}	52.8401	0.159191	newly 5 weeks cumul	47174.	41078.1	{27112., 54328.}	20.7426	0.104817	newly 58 weeks cumul	50734.	48584.9	{30807., 65089.}	23.4533	0.107536
49	newly 3 weeks spec	6054.	3895.69	{2189., 5825.}	44.4553	0.267161	newly 3 weeks cumul	27886.	17075.	{10154., 25287.}	42.8336	0.263363	newly 5 weeks spec	2454.	1810.4	{921., 2687.}	43.2249	0.238992	newly 5 weeks cumul	34341.	21551.9	{12501., 32027.}	42.9486	0.261331	newly 56 weeks cumul	38035.	25146.6	{14380., 37437.}	41.5542	0.246972
50	newly 3 weeks spec	8264.	6282.59	{3310., 9797.}	37.0704	0.202147	newly 3 weeks cumul	33965.	25997.3	{15750., 38346.}	28.5166	0.155538	newly 5 weeks spec	3752.	3063.37	{1406., 4576.}	47.263	0.234577	newly 5 weeks cumul	43375.	33520.5	{19348., 50122.}	31.1708	0.16928	newly 57 weeks cumul	50179.	39398.1	{21816., 58543.}	33.9052	0.180325
